# Expression of Glypican 3 in low and high grade urothelial carcinomas

**DOI:** 10.1186/s13000-015-0266-4

**Published:** 2015-04-21

**Authors:** Oguz Aydin, Levent Yildiz, Sancar Baris, Cihad Dundar, Filiz Karagoz

**Affiliations:** Department of Pathology, Ondokuz Mayis University, Faculty of Medicine, Atakum, Samsun, 55139 Turkey; Department of Public Health, Ondokuz Mayis University, Faculty of Medicine, Samsun, Turkey

**Keywords:** Glypican-3, Urothelial carcinoma, Immunohistochemistry

## Abstract

**Background:**

Glypican-3 (GPC3) is an oncofetal protein which is encoded by GPC3 gene and takes role in the regulation of cell division and apoptosis. Overexpression of GPC3 has been reported in some types of cancer such as hepatocellular carcinoma (HCC), melanoma, squamous cell carcinoma of the lungs and testicular germ cell tumors. The aim of this study was to investigate the immunohistochemical expression of GPC3 in the non-neoplastic urothelium and in urothelial carcinoma (UC). We also aimed to explore the alterations in the GPC3 expression according to the grade and the invasiveness of UC.

**Methods:**

GPC3 expression was studied in 108 UC cases by using immunohistochemistry. Each section was evaluated in terms of the extensiveness and intensity of GPC3 staining. Scores of immunostaining were correlated with tumor grade and stage.

**Results:**

GPC3 expression was observed in 38 cases (35.2%). GPC3 expression was positive in 43.6% of high and in 13.3% of low grade UC (p: 0.003). In 19 UC cases biopsy also harbored non-neoplastic urothelium which showed no staining for GPC3. The difference in staining percentages between low and high grade UCs, suggests that GPC3 staining could be used as an adjunctive marker in cases where the distinction between the low and high grade tumors is difficult. In addition, lack of staining in the non-neoplastic urothelial areas in 19 cases raises the possibility of the use of GPC3 staining for the distinction between neoplastic and non-neoplastic urothelium, especially in punch biopsy samples.

**Conclusions:**

Based on our results potential role of GPC3 in urothelial carcinogenesis warrants further investigation, especially the potential use of GPC3 for therapeutic and diagnostic purposes.

**Virtual Slides:**

The virtual slide(s) for this article can be found here: http://www.diagnosticpathology.diagnomx.eu/vs/2260833001522844

## Background

GPC3 is a cellular surface heparan sulphate proteoglycan which binds to the cell membrane through glycosylphosphatidylinositol anchors [1]. The gene which codes GPC3 is localized in Xq26. GPC3 regulates cell growth and apoptosis by interacting with various morphogenic or growth factors such as Wnt, fibroblast growth factor-2 and bone morphogenic protein [2-4].

GPC3 expression, which is abundant during embryogenesis, is silent in most of adult tissues and therefore it is important as an oncofetal protein [5-7]. Overexpression of GPC3 has been reported in some types of cancer such as HCC, melanoma, squamous cell carcinoma of the lungs and testicular germ cell tumors [8,9].

In HCC, GPC3 stimulates canonical Wnt signaling and enhances in vitro and in vivo tumoral growth. GPC3 is also widely accepted as a tumor marker for HCC [10]. In preliminary studies, GPC3 derived peptide vaccines had favorable effects on the survival in HCC cases [11,12]. Dysregulation of Wnt signaling is proposed to play a key role in the development of UC [13]. It has been demonstrated that Wnt signal was activated in one third of UC samples [14,15]. Therefore, investigation of the role of GPC3 in UC would be interesting both for elucidating of the pathways related to the urothelial carcinogenesis and in terms of demonstration the potential of GPC3 overexpression as a therapeutic target.

In this study we aimed to investigate expression of GPC3 in the non-neoplastic urothelium and in UC by using immunohistochemical methods and to define the alterations in the expression of GPC3 according to grade and invasiveness of tumors.

## Methods

Tissue samples from 108 urothelial carcinoma patients treated in Ondokuz Mayis University Faculty of Medicine between November 2012 and November 2013 were retrospectively analyzed in the pathology department of the institution. Samples of 86 transurethral resections, 12 radical cystoprostatectomies, 7 nephrouretherectomies, and 3 bladder punch biopsies were included in the study. Eleven of the patients were female and 97 were male; the mean age was 68.4 (range 43–92) years. Hematoxylin-eosin sections were re-evaluated and classified according to WHO/ISUP (2004) and staged according to TNM (2009). Informed consent regarding data collection for academic purposes was obtained in all patients. The cases were categorized into 4 major groups as *low grade non-invasive*, *low grade invasive*, *high grade non-invasive* and *high grade invasive*.

### Immunohistochemistry

All sections were examined, and the block that was most representative of the tumor was selected. Four micrometer-thick sections were taken, and immunohistochemical examination was performed with a monoclonal mouse antibody against human GPC3 (1:200, Clone IG12; Cell Marque, Burlington, VT, USA). A standard immunohistochemical technique was performed using a Ventana Benchmark® XT autostainer (Ventana Medical Systems Inc., Tucson, AZ, USA). Appropriate positive and negative controls were included for each run.

Each section was evaluated in terms of the extensiveness and intensity of GPC3 staining. Extensiveness of staining distribution was assessed as percentage of stained cells and was recorded as multiples of ten. A staining percentage ≥ 10% was considered as positive. Staining extensiveness was scored as (1) when 10–50% of the cells were stained, and (2) when > 50% was stained.

GPC3 staining intensity was evaluated only in positive cases and scored as (1) for faint staining (light yellow), (2) for moderate staining (brown), and (3) for strong staining (dark brown). Combined scores were obtained by adding of the intensity and extensiveness scores. Combined score was considered as 0 in the negative cases [16].

### Statistical analysis

The statistical analysis of differences in immunohistochemical staining patterns between groups was performed using Chi square test. Kruskal-Wallis variance test was used in comparison of the scores. Bonferroni-corrected Mann–Whitney *U* test was applied for post hoc binary comparisons. The data was analyzed by the Statistical Package for Social Sciences (SPSS for Windows, version 15.0; SPSS Inc., Chicago, IL). All statistical analyses were two-sided, and p < 0.05 was considered statistically significant.

## Results

Sixty six (61.1%) of the cases were evaluated as invasive, 42 (38.9%) as non-invasive; 78 (72.2%) as high grade and 30 (27.8%) as low grade UC. All of the low-grade cases were non-invasive, while 66 (84.6%) of the high grade cases were invasive and 12 (15.4%) cases were non-invasive (Table 1).

**Table 1:** **GPC3 staining in UC according to grade and invasion**

**Grade**	**No staining**		**Staining**		***X*** ^**2**^	**P**
	**N**	**%**	**N**	**%**		
Low Grade	26	86.7	4	13.3	8.70	0.003
High Grade	44	56.4	34	43.6		
**Invasion**						
Non invasive	31	73.8	11	26.2	2.44	0.118
Invasive	39	59.1	27	40.9		

Positive staining was observed in 38 (35.2%) cases. Staining percentages were 43.6% in high and 13.3% in low-grade UCs. No staining was detected in the non neoplastic urothelium in 19 cases containing non-neoplastic urothelium in the non-tumoral areas.

Percentage of positive staining was found to be significantly higher in high grade than in low grade UC (*X*^2^: 8.70; p: 0.003). Although positive staining was higher in invasive UCs compared to non-invasive UCs, the difference did not reach statistical significance (*X*^2^: 2.44; p: 0.118).

**Figure 1:**
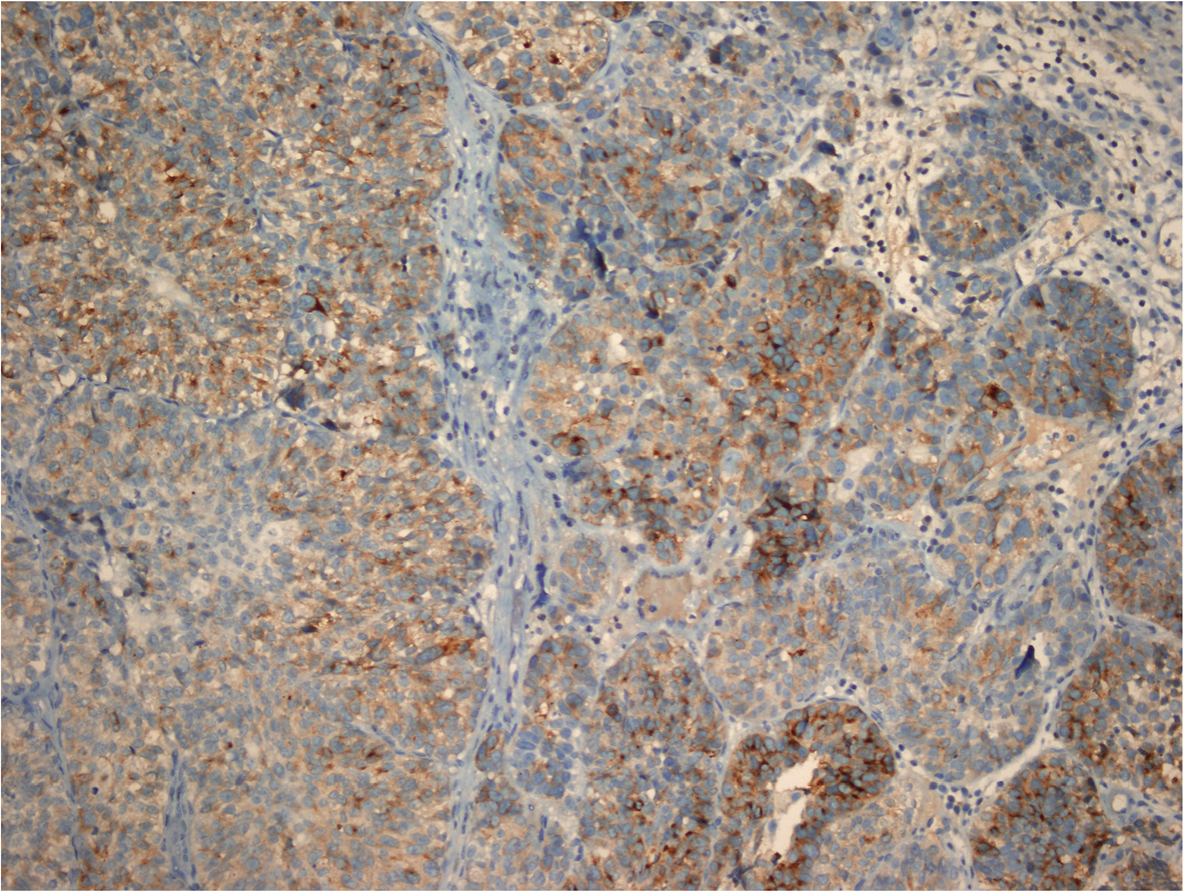
GPC3 staining in high grade invasive UC, with a combined score 5 (immunoperoxidase, x200 magnification).

**Figure 2:**
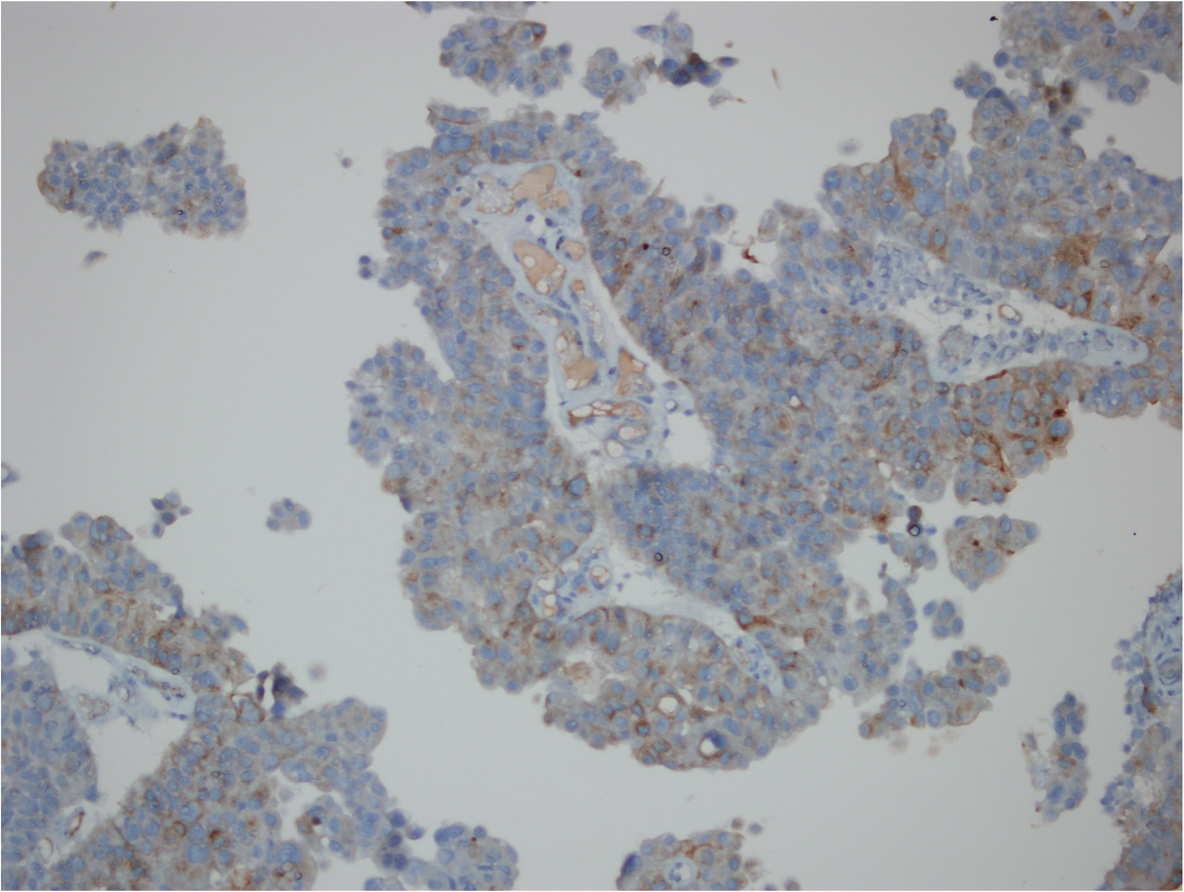
GPC3 staining in high grade non-invasive papillary UC, with a combined score 4 (immunoperoxidase, x200 magnification).

**Figure 3:**
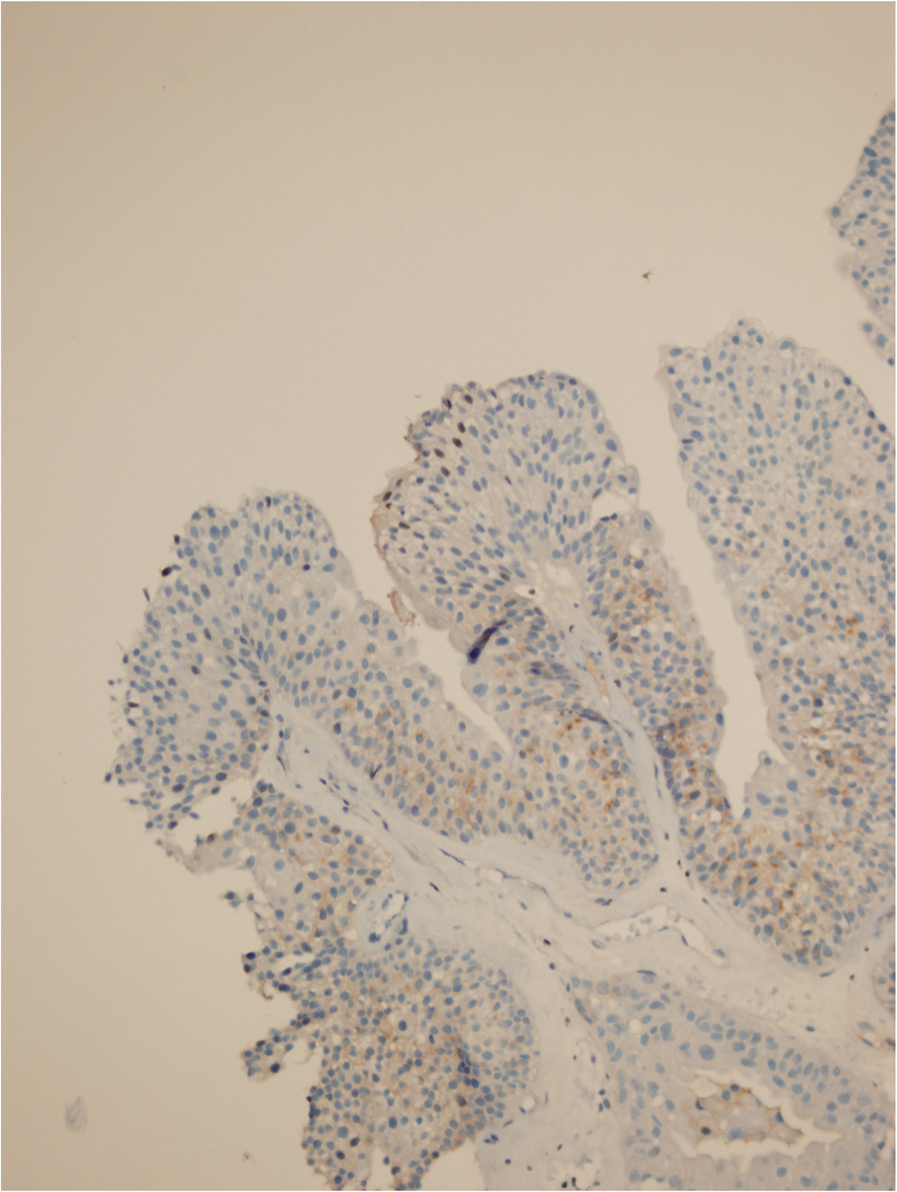
GPC3 staining in low grade non-invasive papillary UC, with a combined score 3 (immunoperoxidase, x200 magnification).

All the extensiveness and intensity scores and combined scores were significantly higher in high grade compared to the low grade UCs (p: 0.004, p: 0.027, p: 0.042, respectively) (Table 2) (Figures 1, 2 and 3).

**Table 2:** **The relationship between the tumor grade and GPC3 staining scores in UC**

**Extensiveness score**	**Low Grade**		**High Grade**		***X*** ^**2**^	**P**
	**N**	**%**	**N**	**%**		
**0**	26	86.7	44	56.4	11.2	0.004
**1**	2	6.7	31	39.7		
**2**	2	6.7	3	3.9		
**Intensity score**						
**0**	26	86.7	44	56.4	9.2	0.027
**1**	1	3.3	4	5.1		
**2**	1	3.3	17	21.8		
**3**	2	6.7	13	16.7		
**Combined score**						
**0**	26	86.7	44	56.4	9.88	0.042
**1**	0	0.0	0	0.0		
**2**	0	0.0	4	5.1		
**3**	1	3.3	16	20.5		
**4**	3	10.0	12	15.4		
**5**	0	0.0	2	2.6

Extensiveness, intensity and combined scores of GPC3 staining was higher in invasive than in non-invasive UCs, but the difference was not statistically significant (Table 3).

**Table 3:** **Correlation between invasiveness and GPC3 staining scores in UC**

**Extensiveness score**	**Non invasive**		**Invasive**		***X*** ^**2**^	**P**
	**N**	**%**	**N**	**%**		
**0**	31	73.8	39	59.1	4.77	0.092
**1**	8	19.1	25	37.9		
**2**	3	7.1	2	3.0		
**Intensity score**						
**0**	31	73.8	39	59.1	2.74	0.434
**1**	2	4.8	3	4.5		
**2**	5	11.9	13	19.7		
**3**	4	9.5	11	16.7		
**Combined score**						
**0**	31	73.8	39	59.1	4.15	0.386
**1**	0	0.0	0	0.0		
**2**	1	2.4	3	4.5		
**3**	4	9.5	13	19.7		
**4**	6	14.3	9	13.7		
**5**	0	0.0	2	3.0		

In the variance analysis carried out in order to test the extensiveness, intensity and combined scores among the groups; a statistically significant difference was found in terms of each three scores (extensiveness score: F: 8.63, p: 0.013; intensity score: F: 9.06, p: 0.011; combined score F: 8.3, p: 0.015). Post-hoc analyzes revealed that the positivity, and the intensity of staining for GPC3 were statistically significantly increased in high grade tumors regardless of the invasiveness of the tumor.

## Discussion

Glypican mediated regulation of signaling requires receptor-ligand interaction and glypicans acts as a stimulator or inhibitory effect on signaling activity [17]. Glypicans also take part in Wnt formation [18,19]. GPC3 is widely expressed during development and is downregulated in most adult tissues [5]. Besides being a tumor marker in hepatocellular carcinomas, GPC3 also plays a role in development and progression and of HCC [10]. Capurro et al. [10], examined the ectopic effect of GPC3 on various cell lines and demonstrated that GPC3 stimulates canonical Wnt signaling, which promotes *in vivo* and *in vitro* HCC growth. Activation of this pathway induces cytosolic accumulation and nuclear translocation of transcription factor β-catenin. In nucleus β-catenin is associated with the members of LEF/TCF transcription factors and induces progression of cell cycle and expression of the genes which stimulate cellular survival [20]. Canonical Wnt activity has been shown to play a role in progression of many cancer types including hepatocellular carcinoma [20,21]. For example, Wnt is active in 90% of the patients with colorectal cancer due to mutations in APC and β-catenin genes, but mutations in these genes are rare in HCC patients despite the fact of existence of the canonical Wnt signal resulting in cytosolic and nuclear accumulation of β-catenin in HCC [22-24]. Thus, overexpression of GPC3 reflects an alternative mechanism in which Wnt activity is stimulated in HCC [25,26]. As in hepatocellular carcinomas, Wnt pathway may be activated by GPC3 in the positive UC cases.

To our knowledge, there are a few studies about GPC3 expression in urothelial carcinomas and normal urothelium [6,27,28]. For example, Baumhoer et al. [6] examined GPC3 expression in the invasive urothelial carcinomas of the bladder using microarray method. They found a positive staining in 7 (16%) of 43 cases. No staining was observed in the normal bladder urothelium. Gailey et al. [27] observed a positive staining for GPC3 in 6 of 49 (12.2%) UC. In both studies, there was no information about the grade and the invasiveness of the tumor. Therefore our study is probably the first study investigating the relationship between GPC3 expression, and the grade and the invasiveness of UCs.

Xylinas et al. [28] studied GPC3 expression in 311 radical cystectomy material. In addition 50 tumor adjacent normal bladder samples were studied as controls. They observed positive staining in 19 (6%) of urothelial carcinomas. The authors underlined that there was no GPC3 expression in benign urothelium.

In our study, GPC3 expression was higher compared to the results obtained in the studies of Baumhoer et al., Gailey et al. and Xylinas et al. [6,27,28]. This is likely caused by the differences in immunostaining techniques. For example the sensitivity of staining may be lower when tissue microarrays with small tissue sections are used [6]. The sizes of the study populations may be another factor. Considering the small number of studies on UCs, more extensive studies including more cases are needed for clarification of the GPC3 expression status and its role in UC. In accordance with the results of the previous studies we did not observe staining in the non-neoplastic urothelium.

Grading and staging of urothelial carcinomas are important since clinical approach changes accordingly. Amongst the non-muscle invading tumors (stages T0-carcinoma in situ and T1) high grade tumors are considered in high risk group. Low risk patients (primary, solitary, Ta, low grade, < 3 cm) will not be treated with adjuvant intravesical BCG, whereas high risk patients will [29]. The difference in staining percentages in low (13.3%) and high grade (43.6%) UCs, suggests that GPC3 staining could be used as an adjunctive marker in cases where the distinction between the low and high grade tumors is difficult. Even more, lack of staining in the benign urothelial areas in 19 cases containing non-neoplastic urothelium, raises the possibility of the use of GPC3 staining for the distinction between neoplastic and non-neoplastic urothelium, especially in punch biopsy samples.

Studies indicate that GPC3 is a promising molecule in immunotherapy [26]. Nakatsura et al. demonstrated in the transgenic rats that GPC3 peptide vaccine induced peptide reactive cytotoxic T lymphocytes without producing autoimmunity. Nakatsura et al. conducted phase 1 clinic trial of a vaccine composed of two GPC3-derived peptides and incomplete Freund adjuvant in advanced HCC patients. The vaccine elicited immune response in the majority of the patients and the level of the immune response was in correlation with the overall survival [11,12,26].

GPC3 expression defined in urothelial carcinomas suggested that GPC3-derived vaccines might have an immunotherapeutic effect on these tumors.

## Conclusion

In conclusion, in our study GPC3 was expressed in a significant proportion of urothelial carcinomas, mostly in the high grade tumors. GPC3 staining may be useful in differentiating between non-neoplastic and neoplastic urothelium as well as high grade and low grade urothelial carcinoma especially in small punch biopsies. Potential role of GPC3 in urothelial carcinogenesis warrants further investigation, especially the potential use of GPC3 for therapeutic and diagnostic purposes.
